# Relationship between a body shape index and muscle strength index in Chinese university students: a cross-sectional survey

**DOI:** 10.1186/s13102-024-00837-1

**Published:** 2024-02-15

**Authors:** Qing Pi, Jiali Xu, Mian Sha, Xiangdong Liu

**Affiliations:** 1 School of Art, Shangrao Preschool Education College, 334000 Jiangxi Shangrao, P. R. China; 2 School of Physical Education, Shangrao Normal University, 334001 Jiangxi Shangrao, P. R. China; 3 Department of Physical Education, Pingxiang University, 337055 Jiangxi Shangrao, P. R. China

**Keywords:** Obesity, Muscle strength, Association, Adolescents, Regression analysis

## Abstract

**Background:**

The problem of overweight obesity and decrease in muscle strength among university students has become an indisputable fact. In this study, a comprehensive index reflecting obesity degree a body shape index (ABSI) and a comprehensive index reflecting muscle strength muscle strength index (MSI) were studied to analyze the cross-sectional correlations existing between them.

**Methods:**

This study began recruiting participants and conducting the test survey in April 2022 and closed in July 2022. Basic condition, height, weight, waist circumference, grip strength, pull-up (boys), bent-leg sit-up (girls), and standing long jump were tested on 12,046 (boys: 6011, 49.90%) university students aged 19–22 years in China, and ABSI and MSI were calculated separately. ABSI was categorized into 5 groups according to age and sex, namely ABSI < 5th percentile (A), 5th ≤ ABSI < 25th percentile (B), 25th ≤ ABSI < 75th percentile (C), 75th ≤ ABSI < 95th percentile (D) and ABSI ≥ 95th percentile (E). The comparison of MSI between different ABSI groups was performed using effect size, and the association between them was performed by curve estimation analysis.

**Results:**

The association between ABSI and MSI of Chinese university students showed an inverted “U” curve. The effect of increased ABSI on MSI was greater in university girls compared to boys. The ABSI of boys was (0.080 ± 0.010) and MSI was (-0.005 ± 2.080); the ABSI of girls was (0.079 ± 0.008) and MSI was (-0.017 ± 1.867). Overall, university students ABSI was at a relatively high point for MSI between 0.050 and 0.100. The university students ABSI at 0.150 had an MSI of -1.229 for boys and − 2.779 for girls.

**Conclusion:**

The ABSI of Chinese university students showed an inverted “U”-shaped curve relationship with MSI, and university students with low or high ABSI had lower MSI. The effect of increasing ABSI on the decrease of MSI was more obvious for girls than for boys.

## Introduction

As the quality of life continues to improve and the level of technology continues to increase, the time of static behavior and video screen time of adolescents continues to increase, and the time of medium and high intensity physical activity decreases, leading to the occurrence of overweight and obesity, and has become a public health problem of global common concern [1, 2]. Studies have confirmed that although the trend of increasing body mass index (BMI) has leveled off among adolescents in developed countries in recent years, it is still on a high rise in developing countries, especially in Asia, and the increase in obesity rate among Chinese adolescents is particularly pronounced [3]. Studies have confirmed that the detection rate of overweight and obesity among Chinese children and adolescents increased from 1.0% to 0.1% in 1985 to 14.0% and 6.4% in 2014, and with the advancement of urbanization in China, the physical fitness of students in both urban and rural areas has also shown a significant downward trend [4, 5].

Currently, most studies use BMI or waist circumference (WC) to measure body fatness [6, 7]. These indicators are widely recognized and adopted because they are simpler and more convenient to test. However, few scholars currently use ABSI, a composite indicator, for body composition evaluation. ABSI is a composite indicator reflecting the proportional relationship between participants’ WC and BMI, which was developed by the study of Krakauer et al [8]. The ABSI index has gradually been widely used by domestic and foreign scholars [9, 10]. Studies have confirmed that ABSI is a more accurate predictor of hypertension and all-cause mortality in adolescents compared to BMI, waist height ratio (WHtR), and WC, and a higher predictor of various cardiovascular diseases and diabetes [11, 12]. Unfortunately, there is less evidence and information from current studies in China using the relationship between ABSI and various physical fitness indicators in adolescents.

The fact that adolescent muscle strength is declining in countries around the world as resistance exercise opportunities in life decrease has been confirmed by several studies [13, 14]. Chinese adolescents are no exception, as Dong et al. confirmed that Chinese adolescents showed a decreasing trend in physical fitness from 1985 to 2014, with a particularly significant decrease in muscle strength [4]. Li et al. also showed a significant decline in muscle strength among adolescents in rural areas of China from 2010–2019 [15]. Previously, there have been more analyses of the causes of muscle strength decline, and most scholars have focused on the study of factors such as lifestyle and physical activity, but rarely analyzed the association that exists between body composition and muscle strength in adolescents.

Past studies have shown that scholars have analyzed the association between body obesity and muscle strength in four main ways. First, most studies have analyzed the association that exists with muscle strength mainly from a single BMI or WC index. For example, Bi et al. analyzed the association between BMI and standing long jump and grip strength in adolescents [16]. Second, most of the past studies analyzed muscle strength using a single indicator to represent muscle strength, such as simply using standing long jump or grip strength indicators to represent muscle strength [17]. Third, past studies on the association between physical obesity and muscle strength have focused on children and adolescents aged 7–18 years, while fewer studies have been conducted on university students who have continued to lose muscle strength. Fourth, most of the previous studies focused on the association between overweight or obesity and muscle strength, but few studies analyzed the association between underweight people and muscle strength.

In view of the limitations of previous studies that mainly existed in the lack of comprehensive indicators reflecting university students’ obesity and muscle strength, and the reality of the continuous decline of university students’ muscle strength in China. To do this, the study surveyed 12,046 university students in China, measuring their body fat and muscle strength. The ABSI, a composite indicator of body obesity, and the MSI, a composite indicator of body muscle strength, were used to analyze the correlation between the two. The hypothesis of this exercise is that both lower and higher ABSIs will negatively impact MSI.

## Methods

### Participants

**Fig. 1:**
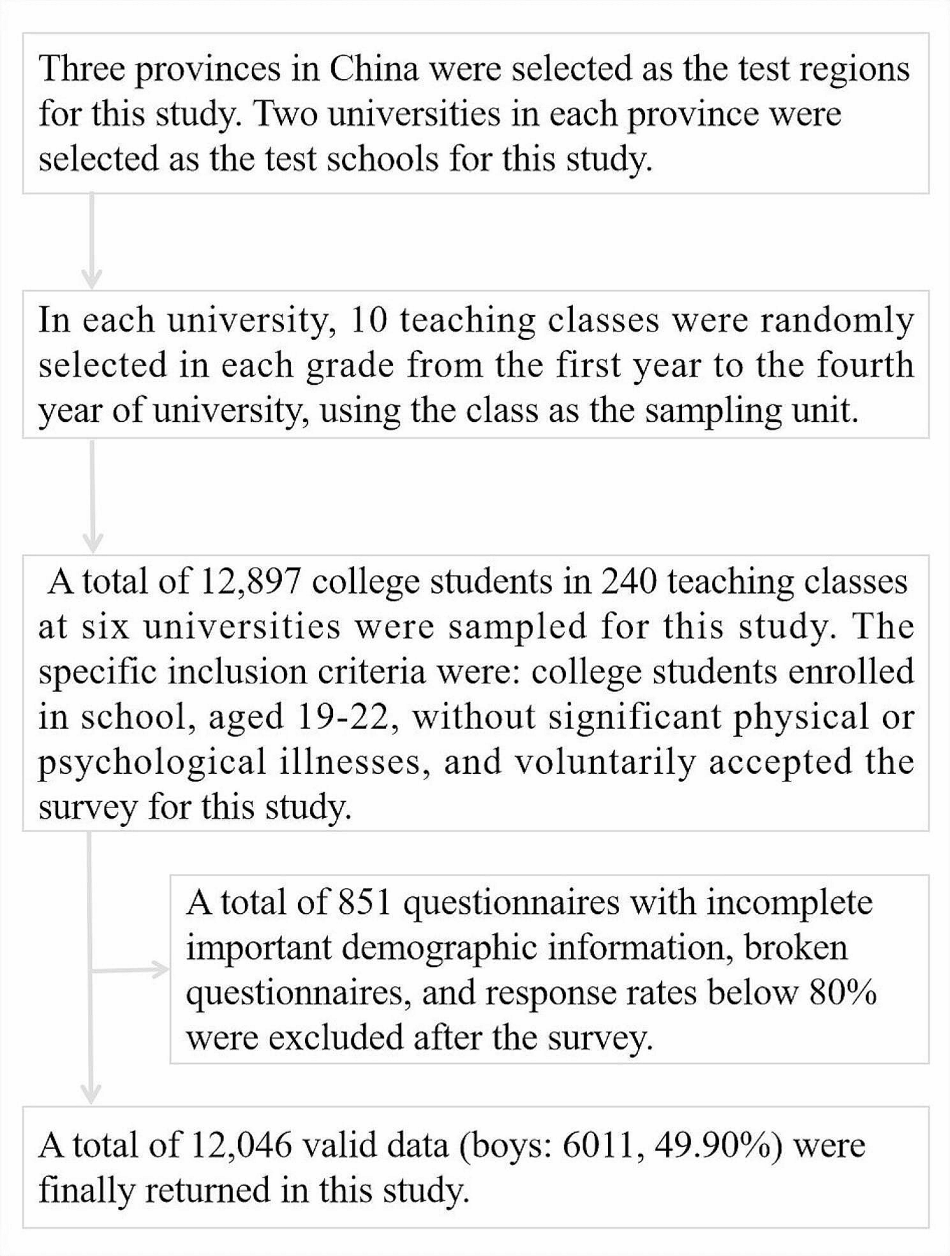
Flowchart of participant sampling of university students in China

A three-stage stratified whole-group sampling method was used to select and survey the participants of this study [18]. This study began recruiting participants and conducting the test survey in April 2022 and closed in July 2022. First, Henan, Jiangxi, and Fujian in China were selected as the test provinces for this study. Second, two universities in each province were selected as the test schools for this study. Third, in each university, 10 teaching classes were randomly selected in each grade from the first year to the fourth year of university, using the class as the sampling unit. A total of 12,897university students in 240 teaching classes at six universities were sampled for this study. The specific inclusion criteria were: university students enrolled in school, aged 19–22, without major physical or psychological illnesses, and voluntarily accepted the survey for this study. A total of 851 survey forms with incomplete important demographic information, broken survey forms, and response rates lower than 80% were excluded after the survey, and 12,046 valid data were finally returned (boys: 6011, 49.90%). The specific sampling process is shown in Fig. 1.

The study was granted with informed consent from the students and parents and written consents were signed. The study was approved by the Ethics Committee of Shangrao Normal University (202,214,045). The survey forms was coded anonymously and the survey forms data were kept strictly confidential to protect the privacy and security of the students.

### A body shape index (ABSI)

ABSI is calculated from BMI and WC by using the formula. the formula for ABSI is WC/(BMI^2/3^×height^1/2^) [8]. BMI is calculated as weight (kg)/height (m)^2^. The unit is kg/m^2^. Height and weight tests are conducted according to the testing instruments and methods specified in the China Student Physical Fitness Survey [18]. The height test was conducted by CSTF-SG3000 height tester, and the test results were accurate to 0.1 cm. The test of weight is conducted by CSTF-TW3000 weight tester, and the test result is accurate to 0.1 kg.

### Muscle strength index (MSI)

The MSI of this study includes a total of three parts of muscle strength indexes of upper limbs, abdomen and lower limbs. The grip strength was used to reflect the upper limb muscle strength of university students. The pull-up (boys) and bent-leg sit-up (girls) were used to reflect the abdominal muscle strength of university students. The standing long jump was used to reflect the muscle strength of the lower limbs of university students. The muscle strength indicators reflecting the three body parts were standardized according to age and gender, and the standardized numbers were summed to obtain MSI. The higher the MSI, the stronger the muscle strength of the participants [19, 20]. The formula for calculating MSI in this study is specifically:

MSI(boys)= Z_grip strength_+Z _standing long jump_+Z_pull−up_

MSI(girls)= Z_grip strength_+Z _standing long jump_+Z_bent−leg sit−up_

The grip strength test was conducted using a MAX EH106 electronic grip strength meter. The participant was asked to use the strong side of the hand to grip the grip strength meter handle, and the number displayed on the display screen was the test result. Participants were tested twice and the highest one was taken as the test result. The results were retained at 0.1 kg [18].

The pull-up (boys) was evaluated by recording the number of tests performed by the subjects university students. The participants were asked to grab the bar with both hands and pull upwards with both hands at the same time, asking the participant’s head to go over the bar, which was recorded once [18]. The number of stretches was recorded by repeatedly following this requirement [18]. The bent-leg sit-up (girls) required participants to lie flat on a sponge mat, bend their legs at 90 degrees at the knees, place their hands with their fingers interlocked behind their necks, and count the number of times their elbows touched their knees, repeatedly. Participants were asked to test as many times as possible within a minute’s time and record the final count [18].

The standing long jump test requires participants to jump forward as far as possible using a standing jump, requiring a two-footed jump [18]. The vertical distance between the line of jump and the nearest heel is recorded. The test results are in centimeters. Each participant was tested twice and the best result was recorded [18].

### Survey of basic information

The survey of basic information mainly included the participants’ region, school, grade, class, college, major, gender, age and other information [18]. The survey of basic information was conducted by means of anonymous numbering.

### Quality control

Participants were required to do preparatory activities before the test to prevent sports injuries during the test. Participants were required to take off their shoes when testing height and to empty their bowels when testing weight, and to wear light clothing for the test [18]. Each program was calibrated by special testing staff before each day’s test to ensure the accuracy of the test. The test conditions were the same for all participants. All participants were tested at the same time. Each participant was tested twice consecutively for grip strength and standing long jump, and once for the other events.

### Statistical analysis

Continuous type variables in this study were expressed as (mean ± standard deviation).

In this study, the calculated ABSI was stratified by percentile. Stratification was based on different age and gender and was divided into five groups: ABSI < 5th Percentile (A), 5th ≤ ABSI < 25th Percentile (B), 25th ≤ ABSI < 75th Percentile (C), 75th ≤ ABSI < 95th Percentile (D), ABSI ≥ 95th Percentile(E). Comparison of MSI between different ABSI groups was performed using effect sizes, and Cohen’s d was divided into small effect = 0.2, medium effect = 0.5, large effect = 0.8 [21].

The association between ABSI and MSI by gender in this study was analyzed using a curve estimation analysis model with the following equations:

Y = aX^2^ + bX + c (Y, MSI; X, ABSI)

Where a (non-linear coefficient), b (linear coefficient), and c (intercept) are constants. Curvilinear regression analysis was performed with Y as the dependent variable and X as the independent variable.

SPSS version 25.0 (IBM, Armonk, NY, USA) software was used for data processing and analysis, and graphs were produced using Graph Pad Prism 8.0.2 (Graph Pad Software, Inc., CA), and the level of statistical significance was set at 0.05.

## Results

Table 1 shows that ABSI for boys was (0.080 ± 0.010) and MSI was (-0.005 ± 2.080); ABSI for girls was (0.079 ± 0.008) and MSI was (-0.017 ± 1.867). In this study, the performance of height, weight, BMI, waist circumference, grip strength, standing long jump, ABSI and MSI were higher for boys than girls.

**Table 1:** Basic demographic information, ABSI and MSI indexes of Chinese university students

Category	Boys	Girls	Total
N	6011	6035	12,046
Age(yr)	20.55 ± 1.07	20.49 ± 1.06	20.52 ± 1.07
Height(cm)	175.39 ± 5.43	163.01 ± 5.01	169.19 ± 8.10
Weight(kg)	70.76 ± 11.17	56.45 ± 8.16	63.59 ± 12.12
BMI(kg/m ^2^)	22.99 ± 3.41	21.23 ± 2.85	22.11 ± 3.27
Waist Circumference(cm)	78.50 ± 9.33	67.10 ± 6.20	72.79 ± 9.75
Grip strength(kg)	45.09 ± 9.08	28.72 ± 6.32	36.89 ± 11.32
Bent-leg sit-up/girls(times)	—	37.20 ± 4.87	37.20 ± 4.87
Pull-up/boys(times)	12.03 ± 4.87	—	12.03 ± 4.87
Standing long jump(cm )	233.17 ± 18.63	173.12 ± 14.32	203.09 ± 34.32
A body shape index	0.080 ± 0.010	0.079 ± 0.008	0.079 ± 0.009
Muscle strength index	-0.005 ± 2.080	-0.017 ± 1.867	-0.011 ± 1.976

Overall, the MSI of the 19-, 20-, and 21-year-old university students 25th ≤ ABSI < 75th Percentile(C) group was the highest, 0.106, 0.018, and 0.027, respectively; the 22-year-old age group university students 5th ≤ ABSI < 25th Percentile (B) had the highest MSI of 0.221. The between-group comparisons of MSI for different ABSI classes university students were made using effect sizes, as shown in Table 2.

**Table 2:** Intergroup comparison of MSI among university students with different ABSI levels in China

Gender/Age(yr)	ABSI<5th Percentile(A)		5th ≤ ABSI<25th Percentile(B)		25th ≤ ABSI<75th Percentile(C)		75th ≤ ABSI<95th Percentile(D)		ABSI ≥ 95th Percentile(E)		Cohen’s d ^※^									
	N	Mean ± SD	N	Mean ± SD	N	Mean ± SD	N	Mean ± SD	N	Mean ± SD	A/B	A/C	A/D	A/E	B/C	B/D	B/E	C/D	C/E	D/E
**Boys**																				
19	61	-0.331 ± 1.956	253	-0.113 ± 1.785	630	0.162 ± 1.982	250	0.105 ± 1.803	62	-0.112 ± 2.000	-0.12	-0.25 ^a^	-0.23 ^a^	-0.11	-0.15	-0.12	0.00	0.03	0.14	0.11
20	82	-0.477 ± 2.583	334	0.131 ± 2.022	827	-0.044 ± 2.034	330	-0.129 ± 1.924	83	-0.338 ± 2.038	-0.26 ^a^	-0.19	-0.15	-0.06	0.09	0.13	0.23 ^a^	0.04	0.14	0.11
21	80	-0.560 ± 1.946	344	0.151 ± 2.276	819	0.026 ± 2.108	331	-0.005 ± 2.128	82	-0.323 ± 2.023	-0.34 ^a^	-0.29 ^a^	-0.27 ^a^	-0.12	0.06	0.07	0.22 ^a^	0.01	0.17	0.15
22	71	-0.250 ± 2.288	292	0.123 ± 2.303	719	0.067 ± 2.191	289	-0.138 ± 2.054	72	-0.363 ± 2.136	-0.16	-0.14	-0.05	0.05	0.02	0.12	0.22 ^a^	0.10	0.20 ^a^	0.11
**Girls**																				
19	64	-0.410 ± 1.436	260	-0.036 ± 2.008	661	0.053 ± 1.703	263	-0.079 ± 2.175	64	-0.351 ± 1.668	-0.21 ^a^	-0.29 ^a^	-0.18	-0.04	-0.05	0.02	0.17	0.07	0.24 ^a^	0.14
20	92	-0.010 ± 1.451	355	0.014 ± 1.902	896	0.075 ± 1.917	359	-0.054 ± 1.844	89	-0.583 ± 1.814	-0.01	-0.05	0.03	0.35 ^a^	-0.03	0.04	0.32 ^a^	0.07	0.35 ^a^	0.29 ^a^
21	81	0.010 ± 1.547	322	0.068 ± 1.873	812	0.028 ± 1.963	317	-0.135 ± 1.973	80	-0.170 ± 1.483	-0.03	-0.01	0.08	0.12	0.02	0.11	0.14	0.08	0.11	0.02
22	66	0.096 ± 1.550	261	0.330 ± 1.867	668	-0.028 ± 1.821	259	-0.338 ± 1.810	66	-0.472 ± 1.578	-0.14	0.07	0.26 ^a^	0.36 ^a^	0.19	0.36 ^a^	0.46 ^a^	0.17	0.26 ^a^	0.08
**Total**																				
19	125	-0.372 ± 1.703	513	-0.074 ± 1.900	1291	0.106 ± 1.845	513	0.010 ± 2.003	126	-0.233 ± 1.836	-0.17	-0.27 ^a^	-0.21 ^a^	-0.08	-0.10	-0.04	0.09	0.05	0.18	0.13
20	174	-0.230 ± 2.070	689	0.070 ± 1.961	1723	0.018 ± 1.975	689	-0.09 ± 1.882	172	-0.465 ± 1.923	-0.15	-0.12	-0.07	0.12	0.03	0.08	0.28	0.06	0.25 ^a^	0.20 ^a^
21	161	-0.273 ± 1.774	666	0.111 ± 2.090	1631	0.027 ± 2.036	648	-0.069 ± 2.053	162	-0.248 ± 1.773	-0.20 ^a^	-0.16	-0.11	-0.01	0.04	0.09	0.19	0.05	0.14	0.09
22	137	-0.083 ± 1.968	553	0.221 ± 2.109	1387	0.021 ± 2.021	548	-0.233 ± 1.943	138	-0.415 ± 1.884	-0.15	-0.05	0.08	0.17	0.10	0.22	0.32 ^a^	0.13	0.22 ^a^	0.10

Note: ^a^ small effect = 0.2, ^b^ medium effect = 0.5, ^c^ large effect = 0.8; N, number; SD, standard deviation; ^※^ Effect size between different groups

**Fig. 2:**
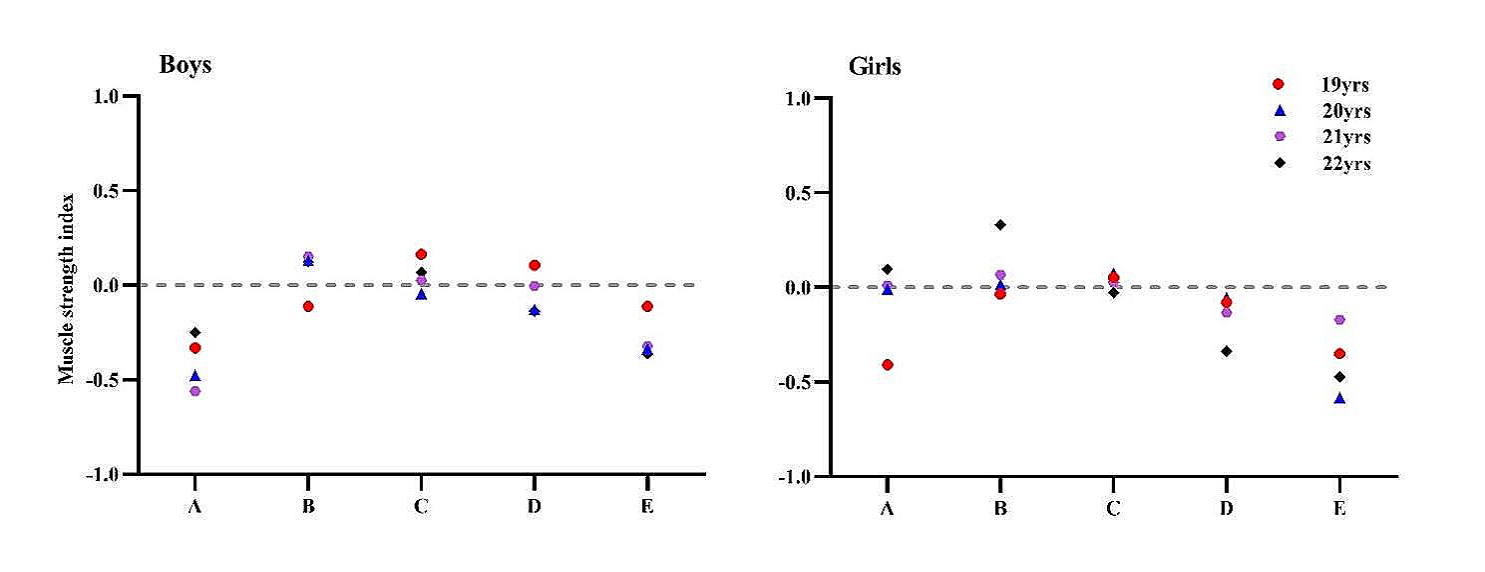
Trends in MSI of university students with different ABSI levels in China. Note: ABSI<5th Percentile(A), 5th ≤ ABSI<25th Percentile(B), 25th ≤ ABSI<75th Percentile(C), 75th ≤ ABSI<95th Percentile(D), ABSI ≥ 95th Percentile(E)

Figure 2 shows a graph of the trend of change in MSI for different ABSI levelsuniversity students in China. Overall, it can be seen that MSI changes with ABSI for both boys and girls in China. Overall ABSI and MSI show a curvilinear relationship, with lower or higher ABSI leading to lower MSI.

This study analyzed gender curve estimation with MSI as the dependent variable and ABSI as the independent variable. Specifically, the following regression equation was obtained:

Boys: Y=-251.534 × ^2^ + 39.940X-1.564 *R*^2^ = 0.002

Girls: Y=-413.763 × ^2^ + 55.411X-1.781 *R*^2^ = 0.004

Total: Y=-254.586 × ^2^ + 36.152X-1.254 *R*^2^ = 0.001

Y, Muscle strength index;X, A body shape index.

**Fig. 3:**
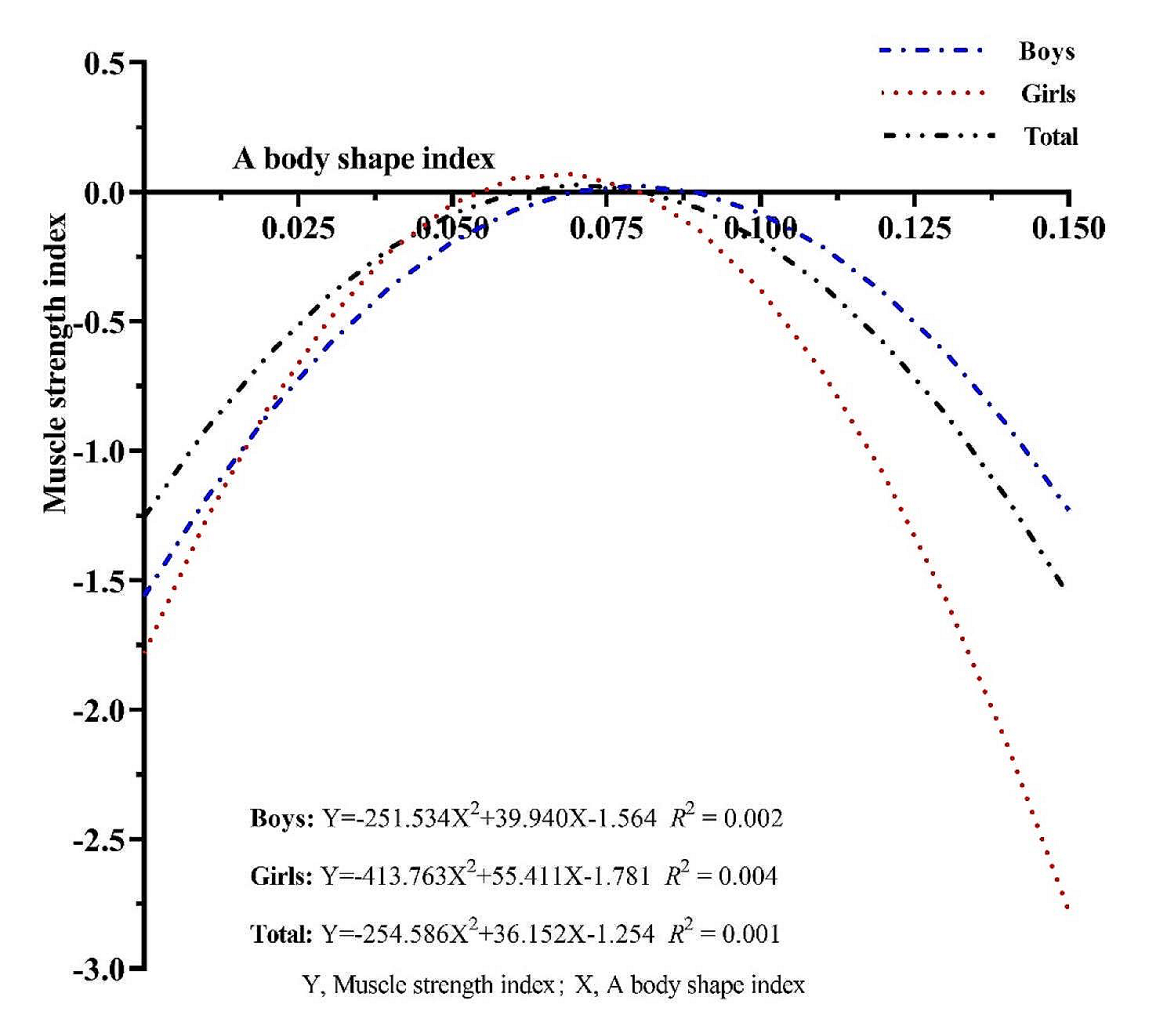
Trend of the association between ABSI and MSI curves for university students in China

From Fig. 3, it can be seen that the ABSI and MSI of Chinese university students show an inverted “U”-shaped curve. In general, the MSI is relatively high when the ABSI of university students is between 0.050 and 0.100. The MSI of university students ABSI is -1.229 for boys and − 2.779 for girls at 0.150. It can be seen that the increase in ABSI of girls has a greater effect on MSI compared to that of boys.

## Discussion

The results of this study showed that the values of ABSI and MSI indicators were higher in boys than in girls in Chinese university students, which is consistent with the findings of related studies [11]. The results of this study further enrich the research on obesity in university students. The reasons for the higher ABSI and MSI indicators for boys than for girls in this study exist in several ways: First, according to the ABSI formula, participants with higher WC values had correspondingly higher ABSI. Studies have confirmed that adolescent boys have higher waist circumference than girls, probably because boys have more bad habits compared to girls, such as excessive alcohol consumption, excessive consumption of sugary drinks, and excessive intake of high-energy meat foods, all of which can lead to a sustained increase in WC, resulting in higher ABSI in boys than in girls [22]. Secondly, university girls pay more attention to their external physical beauty compared with boys, so they often take control of their diet and exercise in their daily life to maintain their external beauty, thus effectively controlling the continuous increase of WC, which may also be an important reason for the lower ABSI for girls compared with boys. The results of this study also showed that MSI was higher in college boys than in girls, which is consistent with the findings of related studies [23–25]. Studies have confirmed that the combined effects of genetic factors, acquired exercise and other factors, the muscle strength of boys is generally higher than that of girls [26, 27]. In addition, university-aged girls are in the late stages of pubertal development, and the secretion of girls estrogen leads to maturation of breast development and an increase in body fat. The need to overcome their own greater body weight resistance when performing muscle strength items such as sit-ups or standing long jump tests may also be an important reason for the lower muscle strength of girls compared to boys in this study.

The results of this study showed that the relationship between ABSI and MSI of Chinese university students showed an inverted “U” curve, indicating that the MSI of university students tended to increase and then decrease as the ABSI increased. This shows that low or high ABSI will have a negative impact on MSI. The results of this study have significant implications for improving muscle strength in college students. Studies have confirmed that adolescents’ BMI and standing long jump show an inverted “U” curve relationship [16]. Li et al. also confirmed that the relationship between university students’ BMI and physical fitness index also showed an inverted “U” curve, in which muscle strength, an important indicator of physical fitness index, also showed the same trend [28]. Chen et al. also confirmed that the BMI of university students showed a “j” curve correlation with body mass index, which is consistent with the findings of this study [29]. Also, other studies have shown that WC also shows a curvilinear relationship with muscle strength indicators, which is consistent with the findings of this study [30, 31].

From the inverted “U” curve of this study, it can be seen that the inverted “U” curve of girls is more “steep” than that of boys. This shows that the increase in ABSI of Chinese girls has a greater effect on MSI than that of boys, i.e., the increase in ABSI leads to a rapid decrease in MSI of girls in university students. This result suggests that we should pay special attention to the continuous increase of ABSI during muscle strength interventions for girls in the future, as long as the ABSI is controlled in a reasonable range to ensure a high level of MSI. This result is consistent with the findings of Bi et al. on BMI and PFI in Xinjiang adolescents [16]. Past studies have shown that girls’ puberty is 1–2 years earlier than boys’, resulting in a stronger correlation between girls’ BMI and physical fitness, which is an important reason why girls’ inverted “U” curve is steeper [32, 33]. Another reason for the steeper inverted “U” curve for girls than for boys may be that the ABSI of girls in universities is relatively low compared to that of boys, and changes in the ABSI of girls can lead to larger changes in the MSI.

There are certain strengths and limitations of this study. The strengths are mainly in two aspects: First, this study is the first to analyze Chinese university students using ABSI, a comprehensive index for evaluating body obesity, and MSI, a comprehensive index for reflecting muscle strength, to more comprehensively respond to the obesity and muscle strength levels of participants. Second, this study surveyed university students in different provinces in China, and the survey sample size was large and representative. However, this study also has some limitations. First, this study was a cross-sectional study, and only the cross-sectional association between ABSI and MSI could be understood, but not the causal association between them. Secondly, this study used epidemiological measurements to judge body obesity, and although a more comprehensive ABSI index was used, there was still some bias compared with the results of measurements such as those using body composition analyzers. These deficiencies need to be remedied in future studies.

## Conclusion

The present study confirmed the inverted “U” curve relationship between ABSI and MSI in Chinese university students, and the effect of increasing ABSI on decreasing MSI was more pronounced in girls students compared with boys students. Based on the results of this study, we suggest that: On the one hand, health education should be provided to university students in the future so that they can develop a healthy lifestyle and ensure that ABSI is in a reasonable range to promote the improvement of MSI. On the other hand, timely exercise and interventions should be carried out especially for obese girls to prevent a drastic decrease in muscle strength. This study also provides a reference and help for future muscle strength interventions for university students.

## Data Availability

No datasets were generated or analysed during the current study.
